# Minimal Inhibitory Concentrations of Thymol and Carvacrol: Toward a Unified Statistical Approach to Find Common Trends

**DOI:** 10.3390/microorganisms11071774

**Published:** 2023-07-07

**Authors:** Barbara Speranza, Antonio Bevilacqua, Daniela Campaniello, Clelia Altieri, Maria Rosaria Corbo, Milena Sinigaglia

**Affiliations:** Department of Agriculture, Food, Natural Resources and Engineering (DAFNE), University of Foggia, 71122 Foggia, Italy; antonio.bevilacqua@unifg.it (A.B.); daniela.campaniello@unifg.it (D.C.); clelia.altieri@unifg.it (C.A.); mariarosaria.corbo@unifg.it (M.R.C.)

**Keywords:** thymol, carvacrol, Minimal Inhibitory Concentration, standardization, trends, dissimilarities, bacteria, fungi

## Abstract

Thymol and carvacrol are some of the most important and used components of Essential oils (EOs); they are widely studied, and there are much data available in the literature. Their Minimal Inhibitory Concentration (MIC) values found in the literature from 2005 to present were used to assess the bioactivity toward yeasts, molds, Gram-positive bacteria, and Gram-negative bacteria, as well as on some bacterial species/serotypes (*Salmonella* sp., *Escherichia coli*, *E. coli* O157:H7, lactic acid bacteria, *Listeria monocytogenes*, *Staphylococcus aureus*, *S. epidermidis*, etc.) to find possible common trends or differences between the two compounds and among the tested species. The results were quite interesting and pointed out that there is a common range for the MIC of thymol and carvacrol for some bacterial species (150–400 mg/L), with some exceptions to this generalized statement. In addition, the statistics pointed out that bacteria could experience homogeneous (*S. epidermidis*, *E. coli* O157:H7) or heterogeneous trends (for example, *Salmonella* sp.) depending on the existence of possible sub-species or different experimental set-ups. Moreover, this paper suggests that there are some drawbacks and issues that should be solved for the effective use of EOs, which are the strong variability among the microorganisms and the lack of standard protocols and reference strains.

## 1. Introduction

Essential oils (EOs) are aromatic and liquid extracts from plant materials, such as flowers, roots, barks, seeds, peels, and fruits [1], that are acquired through steam distillation [2], enfleurage, or expression [3]. They are mixtures of several compounds; some of them are labeled as major if they are present at higher concentrations, ranging from 20% to 70%, while others are considered minor because they are present as traces [4,5]. There are at least 3000 aromatic plants widely distributed in Europe, China, India, Asia, Africa, and South America [6], mainly belonging to Apiaceae, Asteraceae, Lamiaceae, Lauraceae, Myrtaceae, Poaceae, Rutaceae, and Zingiberaceae [7].

The major components of EOs belong to three different classes: terpenes (limonene, α-pinene, p-cymene, etc.), terpenoids (carvacrol, thymol, borneol, and camphor, among others), and phenylpropenes (eugenol, vanillin, and cinnamaldehyde) [7]. It has been reported that the presence of a phenolic ring is a key element for the antimicrobial effect of active components of EOs, as well as methoxy and olefin bonds, as they play a major role in the lipophilicity of the molecules and in their ability to consume proton motive force, affect the intracellular pH value, and disrupt the oxidative phosphorylation of bacteria [8].

The antibacterial effect of EOs has been studied by many authors in the past, and several mechanisms have been demonstrated or postulated, including significant changes in fatty acid profiles, injuries to the cytoplasmatic membrane, reductions in ATP production, increases in membrane permeability, and leakages of some cellular components [4,7,9]. EOs could also play a significant role in fungi inhibition through a variety of mechanisms, like the disruption of inner organelles, mainly mitochondria [10].

Thymol and carvacrol are among the most known and used active components of EOs. Thymol is extracted by many plants, and it is abundantly found in *Thymus vulgaris*, *Ocimum gratissimum*, *Thymus ciliates*, *Satureja thymbra*, *Thymus zygis*, *Trachyspermum ammi*, *Carum copticum*, *Satureja intermedia*, *Thymbra capitata*, *Lippia multiflora*, *Thymus pectinatus*, *Zataria multiflora*, *Satureja hortensis*, *Centipeda minima*, and *Nigella sativa* seeds [11]. It has antimicrobial, anti-inflammatory, antioxidant, antimutagenic, larvicidal, analgesic, and radioprotective effects, among others, and it is generally used in dentistry to counteract oral cavity infections [12].

Thymol is biosynthesized by the hydroxylation of p-cymene through the aromatization of γ-terpinene to p-cymene [13], and no adverse effects are known for its use.

Carvacrol, generally extracted from *Origanum vulgare*, is an isomer of thymol, and its antimicrobial effect is expected to be similar to that of thymol [1]; in addition, a possible anti-biofouling effect, anticancer properties (against breast, lung, and liver cancers), and antioxidant properties were reported [14].

Due to the wide variety of information and data available in the literature, thymol and carvacrol were chosen as the targets for the meta-analysis and statistical evaluation performed in this paper to find possible common trends in the susceptibility/resistance hit for some species or to point out dissimilarities.

The assessment of the bioactivity of an antimicrobial compound generally relies upon the definition of a dose–response profile; the first part of a dose–response profile is the shoulder step, which is a no-effect zone, because the antimicrobial compound does not exert any biocidal/biostatic effect up to a threshold value known as NIC (Not Inhibitory Concentration), which is the highest concentration of the antimicrobial that does not inhibit the studied microorganism [15].

Shoulder is followed by a zone of partial bioactivity, where an increase in the antimicrobial compound causes a reduction in the visible growth of the test microorganisms, up to the Minimal Inhibitory Concentration (MIC), defined as the lowest amount of the studied antimicrobial able to completely inhibit the visible growth of the microorganism [16]. In the zone of partial bioactivity, the antimicrobial does not cause complete inhibition of the test microorganisms and does not exert a biocidal effect.

The definition of MIC is a key step in studying the bioactivity of an antimicrobial compound. There are different approaches, although microdilution and agar diffusion are widespread and the most common protocols worldwide [17]. Most protocols were internally validated by several researchers, but they lack international certification and suffer from several issues, like low sensitivity and the strong effects of several factors (level of the inoculum, temperature, interaction of EOs with the ingredients of the medium, formation of emulsion, low solubility in water, etc.) [18,19]. A proposal for guidelines for EO testing was published in 2021 for lavender, with a focus on all the sources of variability [20].

Another issue for EO and active component bioactivity testing is the lack of reference strains, as researchers usually perform tests and experiments on in-house isolates, many of which are of wild origin. The lack of guidelines and internationally accepted protocols make the comparison of data gained in different countries and laboratories very difficult, as well as the definition of breakpoints and resistance/sensitivity ranges, although interest toward EOs is increasing in the food industry due to consumers’ requests for foods with a lower content of chemical or synthetic additives.

Therefore, this paper proposes a meta-analysis on thymol and carvacrol, focusing on MIC values found in the literature on several bacterial and fungal species, mainly of foodborne origin, to find common trends in the bioactivity of these compounds and to point out possible ranges or breakpoints for an effective application in a wide variety of conditions, as well as to highlight similarities and dissimilarities between them and among the tested species, as preliminary steps for the development and validation of an internationally recognized protocol and a set of guidelines and reference breakpoints to study their antimicrobial effect.

## 2. Materials and Methods

### 2.1. Data

The data used in this manuscript are MIC values of thymol [5-methyl-2-(methylethyl)phenol] and carvacrol [2-methyl-5-(methylethyl)phenol], available in the literature in research papers from international databases, Scopus and PubMed. The keywords for search were “thymol”, “carvacrol”, “antimicrobial effect”, “Minimal Inhibitory Concentration”, and other similar words. After this first step, ca. 4000 articles were found; to select the most relevant ones, only research papers that fit the following inclusion criteria were used:

(a) Papers from 2005 to present.
(b) Use of collection isolates or isolates that had been previously identified through genotyping; papers without a clear identification of the strains or with strains identified only through phenotyping were excluded.
(c) Assays performed in the optimal broth for each microorganism, not containing restrictive ingredients or conditions (antibiotics, salt, or sub-optimal pH), which could affect the response of the test strains to carvacrol and thymol.
(d) Use of thymol and carvacrol with a degree of purity of at least 96%.
(e) Only research papers with a clear distinction between MIC and MBC (minimum bactericidal concentration) were used. The articles where the threshold values of thymol and carvacrol could be either MIC or MBC were excluded.
(f) Use of both positive (for example, chloramphenicol, cycloheximide, or other antibiotics able to exert inhibition against the tested microorganism) and negative controls (distilled water and the solvent used to prepare thymol and carvacrol solution). Research papers not containing negative controls were excluded.
(g) Experiments conducted using the micro-dilution approach; it was not possible to include papers that used CLSI protocols, as they are generally used for antibiotics, and only in recent years has a proposal for a protocol for EO_S_ been released.
(h) For the analyses, some papers with an agar diffusion approach were retained if the experiments had been conducted with at least 10 different concentrations of thymol and carvacrol and positive and negative controls were obtained, as reported for point “f”.
(i) Only species (bacteria or fungi) for which there were at least three independent evaluations of MICs were retained for this study.

The dataset produced and used for statistics is in supplementary Table S1.

### 2.2. Standardization and Data Analysis

The data reported in Table S1 were preliminary converted into mg/L and then into natural logarithm. A first classification was conducted for macro-categories, as MIC values were divided into four classes (Gram-positive bacteria, Gram-negative bacteria, yeasts, and molds), analyzed using the non-parametric tests of Mann–Whitney and Kruskal–Wallis (critical P set at 0.05) and the outlier test, and graphically reported through a box–whisker plot, where the box represents the interquartile range and the whisker represents the range from minimum to maximum.

A second classification was conducted for Gram-positive and Gram-negative bacteria, focusing on species/serovar level. The classes for Gram-positive bacteria were *Staphylococcus aureus*, *Staphylococcus epidermidis*, *Listeria monocytogenes*, *Bacillus cereus*, *Bacillus subtilis*, other *Bacillus* spp., enterococci, lactobacilli, and streptococci, while the groups for Gram-negative were *Escherichia coli*, *E. coli* O157:H7, *Salmonella* sp., other enterobacteria, *Pseudomonas* spp., and *Proteus* spp. The differences amongst the different groups of Gram-positive or Gram-negative bacteria were analyzed using Kruskal–Wallis test, while data were presented in box–whisker plots.

As a second step, data for some species (*S. aureus*, *S. epidermidis*, *Salmonella* sp., *E. coli* non O157:H7, and *E. coli* O157:H7) for both thymol and carvacrol were analyzed through PERMANOVA (permutational analysis of variance), using the antimicrobial compounds and the species as categorical predictors, with critical P set to 0.05; the post hoc multiple comparison was conducted using the Mann–Whitney pairwise test. On the same data, a violin graph was built for each combination microorganism/antimicrobial compound, thus gaining 10 different probability distributions.

Statistics were obtained using the software Statistica for Windows ver. 7.0 (Statsoft, Tulsa, OK, USA) or PAST free software ver. 4.2 (Natural History Museum, University of Oslo, Oslo, Norway).

## 3. Results and Discussion

EOs, as well as their active compounds, were tested toward a wide range of microorganisms, both in laboratory media and in foods [8,21]; however, the lack of a standardized protocol could be an issue for the definition of sensitive and resistant species, as well as for the definition of a susceptibility hit [22]. Several variables could strongly affect the bioactivity of EOs [1,4,23]; among others, the protocol for their extraction is an issue. In addition, the effects on microorganisms could rely on both the main components and minor ones, as some moieties, when present in traces, could strengthen the bioactivity of an oil [4,7,8].

As a rationale, this paper focuses on two representative compounds, thymol and carvacrol, with a well-defined chemical structure, and whose chemistry is well known to avoid any confounding effect related to EO composition. They have a core phenolic ring in their moieties, and an OH-secondary group, although in different positions, similar densities, and boiling points (0.969 vs. 0.973 mg/L for the density and 233 °C vs. 237 °C for the boiling points) [11,14]. The choice of isomers with similar properties also avoids the cofounding effect related to chemistry. In the following sections, each compound is separately analyzed using non-parametric tests and box plot graphs; namely, the MIC values were analyzed according to different categories. First, the analysis was conducted using Gram-positive or Gram-negative bacteria, yeasts, and molds as predictors. Then, a focus was conducted within Gram-positive or Gram-negative bacteria to point out similarities or dissimilarities. For each category, some representative species were chosen, that is, species for which a high number of MIC values were found in the literature. For this approach, box plot figures were built using the interquartile range for the box and the median as the central point, thus showing the MIC range of thymol and carvacrol for 50% of the strains/species analyzed for each group; moreover, whisker was conducted with theoretical minimum and maximum values, that is, excluding the outliers, thus pointing out the possible range inside which MIC values could be considered typical of the species.

In the last step, a comparative analysis of thymol and carvacrol was conducted, along with a focus on the probability distribution of the data, to point out differences between the two compounds and the characteristic trend (if any) for some species.

### 3.1. Thymol

**Figure 1:**
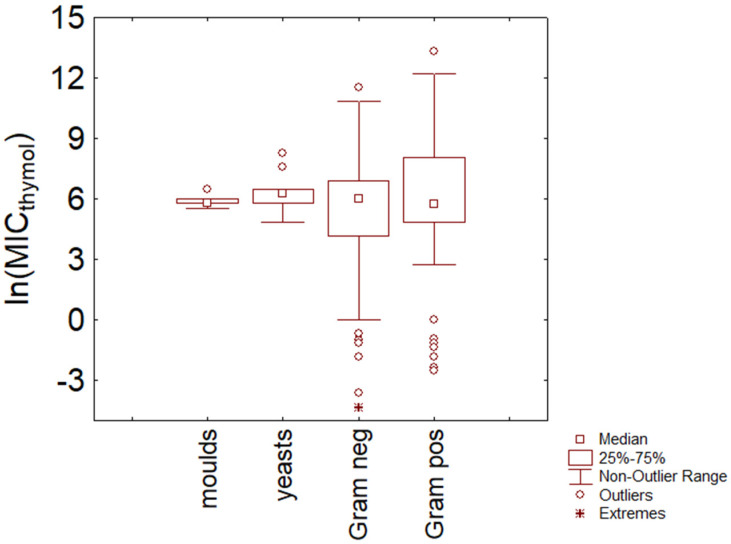
Minimal Inhibitory Concentration (MIC) of thymol (reported as natural logarithm of actual amounts in mg/L) toward Gram-positive and Gram-negative bacteria, yeasts, and molds.

Figure 1 shows a box plot diagram for all the microorganisms, classified into four main categories: molds, yeasts, Gram-negative, and Gram-positive microorganisms; all data were preliminary standardized as ln (concentration). Median values were 5.76 ln (mg/L) for molds, 6.24 ln (mg/L) for yeasts, 5.99 ln (mg/L) for Gram-negative microorganisms, and 5.76 ln (mg/L) for Gram-positive microorganisms, corresponding to actual values of 317, 513, 400, and 317 mg/L; the differences amongst the categories were not significant (P, 0.66 for Kruskal–Wallis test). The figure also points out the strong variability for the dataset of bacteria, as suggested by the quartiles and minimum and maximum values.

For Gram-negative bacteria, the first and third quartiles were, respectively, 4.13 and 6.90 ln (mg/L), thus highlighting that 50% of Gram-negative microorganisms showed a MIC value for thymol ranging from 60 mg/L to 900 mg/L. The statistics also pointed out the theoretical minimum and maximum values of the distribution (0 and 10.8 ln (mg/L), i.e., 1 and 49,000 mg/L), which further highlight the strong variability of the dataset. In the case of Gram-positive bacteria, the first and third quartiles were 4.85 and 8.06 ln (mg/L) (127–3165 mg/L), while the theoretical minimum and maximum of the distribution were 2.76 and 12.12 ln (mg/L) (16–183,505 mg/L).

In the past, many authors have reported a higher sensitivity of Gram-negative microorganisms than Gram-positive bacteria, and this difference has been generally attributed to the peptidoglycan of Gram-positive microorganisms and the higher resistance that it exerts toward hydrophobic compounds [24,25,26]. The data of this meta-analysis do not confirm this trend, probably depending on the high variability inside the dataset but also on the use of a wide range of microorganisms rather than a single strain. This latter idea confirms the hypothesis that in some situations, the choice of the incorrect type or test strain could lead to a systematic bias.

In addition, the difference in the bacterial resistance to EOs is a matter of debate, as other authors have not reported any differences [27,28]. As correctly stated by Angan et al. [29], the protocols for laboratory testing could strongly affect the results, and the choice between the agar-diffusion and micro-dilution method could lead to differences or not, depending on the ability of hydrophobic EOs to enter into contact with microorganisms.

**Figure 2:**
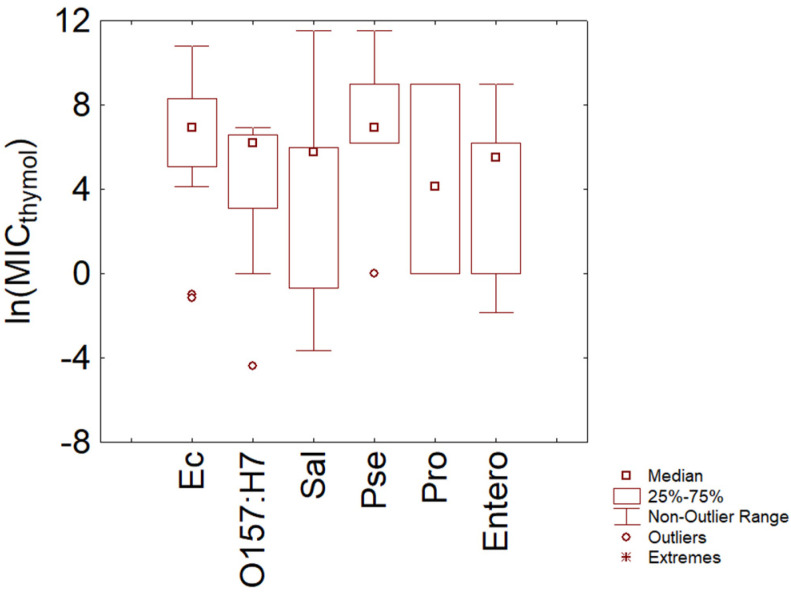
Minimal Inhibitory Concentration (MIC) of thymol (reported as natural logarithm of actual amounts in mg/L) toward Gram-negative bacteria. Ec, *E. coli*; O157, *E. coli* O157:H7; Sal, *Salmonella* sp.; Pse, *Pseudomonas* spp.; Pro, *Proteus* spp.; Entero, other enterobacteria.

After the analysis on the data related to the macro-categories, MICs were separately analyzed for Gram-negative and Gram-positive bacteria to point out possible species differences. Figure 2 shows the box plot for Gram-negative species for which 6–10 different MIC values were found, that is, *Escherichia coli* (separately analyzed for O157:H7 serotype and other *E. coli*), *Salmonella* sp., *Pseudomonas*, *Proteus*, and other enterobacteria. In particular, the species (or the serotypes) with the highest number of input values were *E. coli* O157:H7, other *E. coli*, and *Salmonella* sp., with median values of 6.91, 6.21, and 5.76 ln (mg/L) (1000, 498, and 317 mg/L), thus pointing out some dissimilarities between the serotype O157:H7 and the other *E. coli*, as well as between *E. coli* species and *Salmonella* sp. However, the groups also showed a strong variability in their dataset.

For *E. coli* O157:H7, the first and third quartiles were 4.66 and 6.38 ln (mg/L) (106 and 590 mg/L), while the theoretical minimum and maximum MIC values were set by the statistics to 0 and 6.9 ln (mg/L), with some outliers below the minimum, which suggests that for some analyses, some authors used strains, which could not be considered a good test for this serotype, or the application of protocols, which could lead to bias. For the other *E. coli* strains, the statistics suggest a trend toward a MIC increase, as the first and third quartiles were 5.08 and 8.15 ln (mg/L) (161 and 3460 mg/L), while the minimum and maximum of the distribution were set by the statistics to 4.1 and 10.89 ln (mg/L) (60.34 and 53,100 mg/L).

A high variability was also found for *Salmonella* sp.; the first and third quartiles for this species were 0.69 and 5.99 ln (mg/L) (corresponding to actual values ranging from 0.5 to 400 ppm), while the extremes of the statistical distribution were set at −3.9 and 11.6 ln (mg/L).

Although the dataset had fewer values, the statistics also confirmed the high resistance of *Pseudomonas* spp., which showed a median MIC of 6.90 ln (mg/L) and quartiles at 6.21 and 8.99 ln (mg/L) (respectively, 992, 498, and 8000 mg/L).

**Figure 3:**
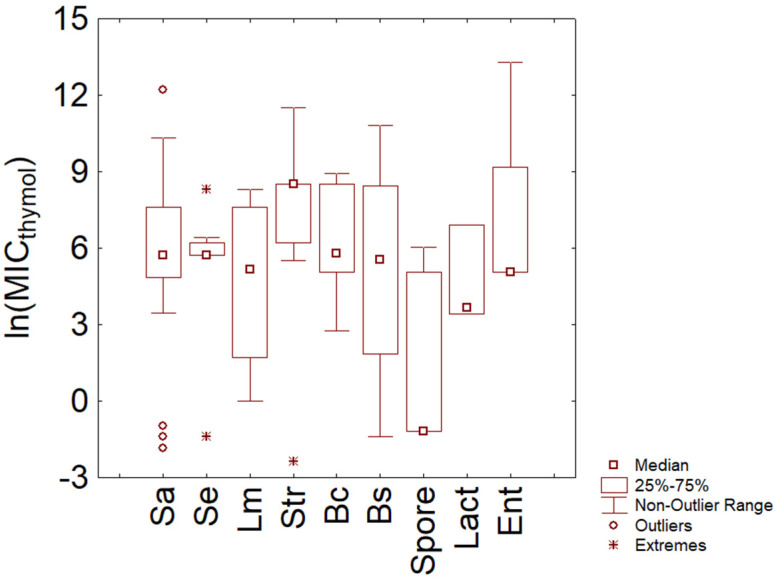
Minimal Inhibitory Concentration (MIC) of thymol (reported as natural logarithm of actual amounts in mg/L) toward Gram-positive bacteria. Sa, *S. aureus*; Se, *S. epidermidis*; Lm, *L. monocytogenes*; Str, streptococci; Bc, *B. cereus*; Bs, *B. subtilis*; spore, other spore-forming bacteria; Lact, lactobacilli; ent, enterococci.

The last analysis conducted for thymol was on Gram-positive bacteria (Figure 3); most data were found for *Staphylococcus* spp. (*S. aureus* and *S. epidermidis*) and streptococci. The plot points out the different trends for the two species of the genus *Staphylococcus*, as *S. epidermidis* showed a lower variability in MIC distribution (median 5.73 ln (mg/L), and interquartile range 5.73–6.2 ln (mg/L), corresponding to actual values of 308–493 mg/L). In the case of *S. aureus*, although the median was similar (5.73 ln (mg/L)), the MIC distribution was characterized by higher variability, as one could infer from the interquartile range (4.85–7.60 ln (mg/L), that is, 128–2000 mg/L) and minimum and maximum of the distribution (3.2–10.7 ln (mg/L)).

Streptococci showed a generally higher MIC (Kruskal–Wallis, *p* < 0.5), with a median value of 8.52 ln (mg/L) and an interquartile range of 7.25–8.52 ln (mg/L) (actual values between 1400 and 5000 mg/L). Finally, the MIC medians for lactobacilli, enterococci, *L. monocytogenes*, *B. cereus*, and *B. subtilis* were 3.67, 5.08, 5.17, 5.79, and 5.65 ln (mg/L) (39.25, 161, 176, 330, and 284 mg/L, respectively). Amongst the Gram-positive microorganisms, lactic acid bacteria seemed to be more resistant, and the data found partly confirms this trend, at least for streptococci [30].

### 3.2. Carvacrol

**Figure 4:**
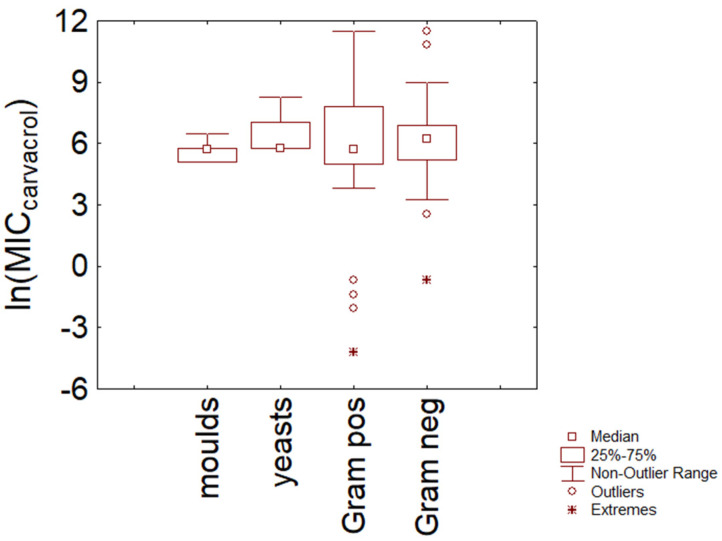
Minimal Inhibitory Concentration (MIC) of carvacrol (reported as natural logarithm of actual amounts in mg/L) toward Gram-positive and Gram-negative bacteria, yeasts, and molds.

The same approach was used to gain details and information on the quantitative distribution of MIC values of carvacrol. Figure 4 shows the trend for the macro-categories (molds, yeasts, Gram-negative and Gram-positive bacteria). Bacteria showed a higher variability than fungi, but this result could also be due to the lower observations available for molds and yeasts; namely, for Gram-negative bacteria, the median of MIC values was 5.74 ln (mg/L) with an interquartile range of 5.01–7.82 ln (mg/L) (311 and 150–2490 mg/L). The statistics also predicted the minimum and the maximum of the MIC distribution at 3.9 and 11.5 ln (mg/L) (49.40–100,000 mg/L). In the case of Gram-positive bacteria, MIC values were predicted to be in the range 3.2–8.9 ln (mg/L) (minimum and maximum), with a median value of 5.73 ln (mg/L) (45–7330 and 308 mg/L, respectively); MIC median values for fungi were 5.74–5.77 ln (mg/L) (ca. 300 mg/L).

**Figure 5:**
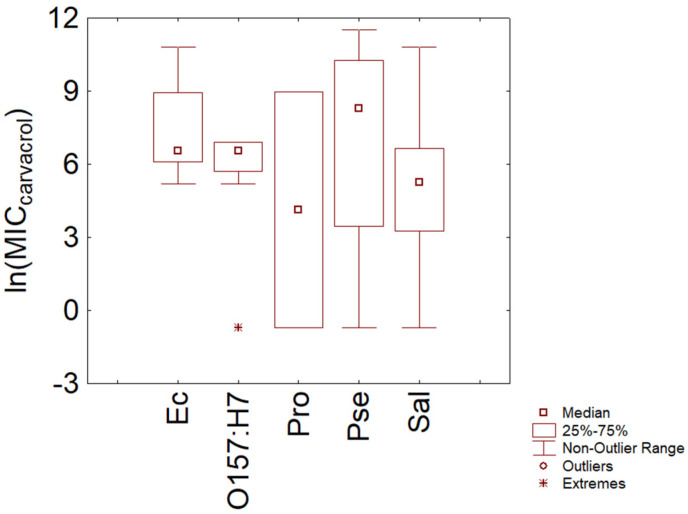
Minimal Inhibitory Concentration (MIC) of carvacrol (reported as natural logarithm of actual amounts in mg/L) toward some Gram-negative bacteria. Ec, *E. coli*; O157:H7, *E. coli* O157:H7; Pro, *Proteus* spp.; Pse, *Pseudomonas* spp.; Sal, *Salmonella* sp.

A focus on the MIC distribution at the species level was also possible for some Gram-negative and Gram-positive microorganisms. Concerning Gram-negative bacteria (Figure 5), the box plot suggests a similar trend for *E. coli* O157 and other *E. coli* (median MIC at 5.76 ln(mg/L)), although non-O157 strains showed a higher variability, with an interquartile range of 6.13–8.77 ln (mg/L) (459–6400 mg/L).

A high variability was also found for *Salmonella* sp., which showed a median MIC of 5.27 ln (mg/L) and an interquartile range of 3.26-6.66 ln (mg/L) (194 and 26–780 mg/L).

**Figure 6:**
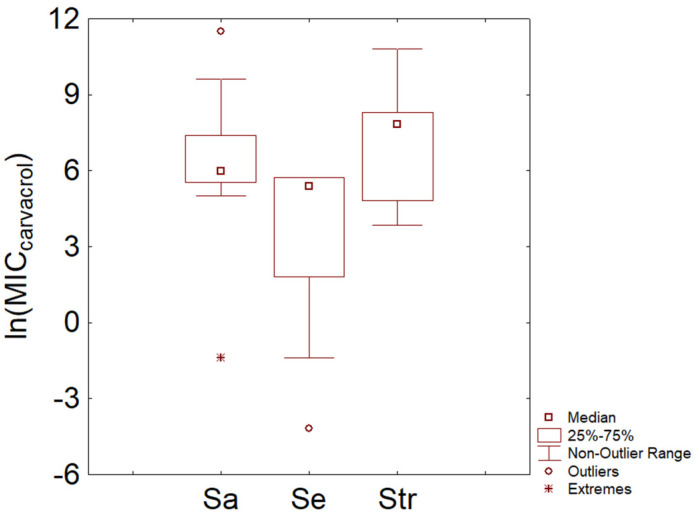
Minimal Inhibitory Concentration (MIC) of carvacrol (reported as natural logarithm of actual amounts in mg/L) toward some Gram-positive bacteria. Sa, *S. aureus*; Se, *S. epidermidis*; Str, streptococci.

Some Gram-positive species are shown in Figure 6. The MIC distribution was built only for *S. aureus*, *S. epidermidis*, and streptococci due to a relatively higher number of cases. For *S. aures*, the median value was 5.99 ln (mg/L) (399 mg/L), while *S. epidermidis* showed a trend toward a lower MIC (median at 5.37 ln (mg/L) and interquartile at 3.41–5.74 ln (mg/L), that is, 215 and 30.27–311 mg/L). For streptococci, the MIC median was 7.82 ln (mg/L), with an interquartile range of 6.33–8.29 ln (mg/L) (2489 and 561–3983 mg/L).

### 3.3. Comparison

As a second step, a PERMANOVA analysis was conducted on some representative species (*S. aureus*, *S. epidermidis*, *E. coli*, *E. coli* O157:H7, and *Salmonella* sp.) for which a high number of MIC values had been recovered. For this analysis, two predictors were used: the antimicrobial compound (thymol or carvacrol) and the bacterial species. PERMANOVA is a non-parametric approach similar to multivariate ANOVA and is useful to point out the effect of two or more predictors on a dependent variable. Its outputs are the sum of squares, the mean square (that is, the sum of squares standardized to the degree of freedom), the Fisher test (standardized weight of each predictor), and the P-value.

The only significant predictor was the species, while the kind of antimicrobial and the interactive term (antimicrobial compound vs. bacterial species) were not significant (Table 1).

Significant differences were found between *S. epidermidis* and *E. coli* (Mann–Whitney test, *p* < 0.01) and between *S. epidermidis* or *E. coli* O157:H7, while the effect of the kind of antimicrobial compound was not significant for any of the tested species (*p* > 0.05). The lack of differences between thymol and carvacrol was an expected result, as the two compounds are isomers; they have a phenolic ring and a secondary -OH group, although in different positions. These elements could play a major role in the bioactivity of compounds of natural origin, although the position of the secondary group could also cause some differences in the bioactivity between carvacrol and similar compounds lacking the hydroxyl group, like carvacrol methyl ester, methanol, and cymene [23], as evidenced in the case of carvacrol and by the differences. There are conflicting data in the literature, as some authors reported similar MIC values for these two compounds, for example, against *E. coli* (200 mg/L), *S. aures* as planktonic cells or biofilms (0.01–0.03%), or *S. epidermidis* (0.03–0.12%) [31,32]; other authors reported a higher bioactivity of the carvacrol, for example, against *Ps. aeruginosa* [23]. However, it is worth mentioning that the cited authors performed the experiments on a single strain, and the results were probably affected by strain variability and protocol conditions.

**Figure 7:**
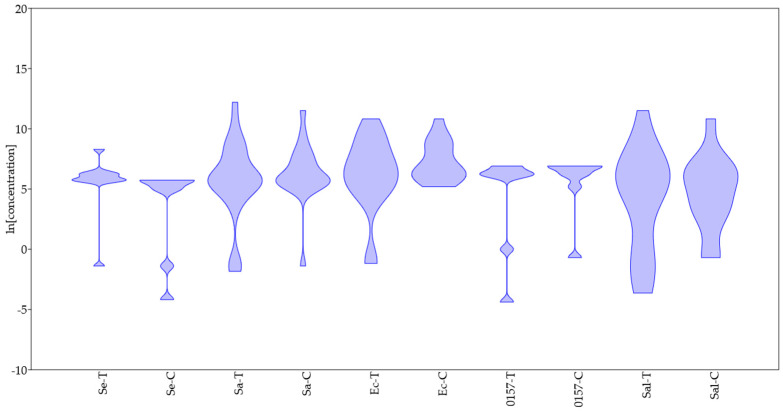
Violin plot for MIC values of thymol (T) and carvacrol (C) toward *S. epidermidis* (Se), *S. aures* (Sa), *E. coli* (Ec), *E. coli* 0157:H7 (O157), and *Salmonella* sp. (Sal).

As a final step for these representative species, a violin plot was conducted (Figure 7). This approach combines the classical box plot with a probability distribution. It is a powerful tool for the visualization of data from both small-size and high-size samples; moreover, it enables the reader to understand whether the data distribution follows a normal trend or if it is unimodal (with a single value as the mode) or multimodal (with two or more values considered the mode). None of the studied species showed a normal distribution for MIC values of both carvacrol and thymol, and generally, the mode was in the range 5–6 ln (mg/L), thus suggesting that most strains had real MIC values of thymol and carvacrol ranging from 150 to 400 mg/L. In addition, the violin plot shows the existence of possible sub-species or of different sensitivity profiles depending on different experimental approaches, for example, in the case of *S. aureus* for thymol, with most strains with an MIC value of 5 ln (mg/L) (150 mg/L) and a few strains with a very sensitive trend and an MIC of −1 or −2 ln (mg/L) (0.13–0.37 mg/L). Possible sub-classes were also pointed out in the case of *E. coli* again for thymol: most strains had a resistant trend (MIC > 5 ln (mg/L)) and a few strains had a sensitive trend (MIC at −1 ln (mg/L)). The existence of classes or a strong variability amongst the strains of a single species could be an issue, as it suggests an unpredictable use for practical applications (foods or clinical uses, among others), as also suggested for other natural products [22].

For other combinations of antimicrobial compounds/microorganisms, the species showed a homogeneous trend with a strict range of values for the MIC, along with some abnormalities in the distribution, which are probably related to methodological issues or to the use of sensitive strains; this kind of distribution was found for *S. epidermidis* or *E. coli* O157:H7 for both thymol or carvacrol, thus suggesting the possibility of pointing out well-defined ranges of concentrations of thymol or carvacrol that are useful for antimicrobial purposes. For other species (for example, *Salmonella* sp.), the distribution was not homogeneous, and the strains belonging to them could experience heterogeneous trends with strong intra-species variability, suggesting the need for a preliminary screening to point out the concentrations of thymol and carvacrol exerting an antimicrobial action.

## 4. Conclusions

EOs could be promising alternatives to inhibit a wide range of microorganisms, both for food preservation and for clinical uses. The survey of the literature and the analysis of the MIC values of thymol and carvacrol suggest that there are some issues that should be addressed before an effective scaling up of these compounds can take place. The most important could be the strong variability among the microorganisms and the lack of standard protocols and reference strains.

Despite the issues and limitations of using data produced by different researchers and authors with very different experimental approaches, this paper could offer a contribution to the debate on the bioactivity of EOs. First, it confirms the strong variability in the resistance/susceptibility profiles of bacteria and fungi and the existence of differences among strains of the same species; however, it also offers novel points, perspectives, and ideas for future research. The most important key-points could be summarized as follows:

(a) There is no evidence of a different resistance between Gram-positive and Gram-negative bacteria and fungi, at least for thymol and carvacrol and at least for the data used in this paper. Some differences were found at the species level (for example, between *S. epidermidis* and *E. coli*).
(b) The probability distribution found through the violin plot pointed out a possible homogeneous trend toward resistance to thymol and carvacrol for some species (*S. epidermidis*, *E. coli* O157:H7), while for other species (for example, *Salmonella* sp.), the trend was strongly variable, and this could be an issue to think for an effective industrial use of some Eos.
(c) Some species could show different profiles of resistance/sensitivity, as suggested, for example, for *E. coli* in the case of thymol. An open question is whether these different profiles depend on the existence of sub-species or on different experiment set-ups.
(d) There is a well-defined range for thymol and carvacrol concentrations (5–6 ln (mg/L); 150–400 mg/L), where the probability of finding MIC for many species is high.
(e) Finally, the statistical treatment of data is crucial, as most of the evidence and new perspectives in this paper were found only after a standardization of the data as a natural logarithm and using both non-parametric tests and probability distributions (i.e., a violin graph).

There is a lot of work to do in the field of the antimicrobial use of EOs, mainly in terms of protocol standardization, definition of thresholds, and guidelines, which are fundamental steps to gain robust data and build robust sensitivity/resistance profiles. This paper wants to offer a contribution to this context, also proposing possible approaches for data treatment and their standardization.

## Figures and Tables

**Table 1:** PERMANOVA run on MIC values of *S. epidermidis*, *S. aureus*, *E. coli*, *E. coli* O157:H7, and *Salmonella* sp. Species, effect of the kind of microorganisms; antimicrobial, effect of thymol and carvacrol; F, Fisher test; sum of sqrs, sum of squares; df, degree of freedom.

Predictor	Sum of Sqrs	df	Mean Square	F	*p*
Species	103.85	4	25.96	2.32	0.03
Antimicrobial	4.80	1	4.80	0.43	0.48
Interaction	−133.69	4	−33.42	−2.99	0.31
Residual	1452.10	130	11.17		
Total	1427.10	139			

## Data Availability

Data used for this paper are available in the Supplementary Materials.

## Supplementary Materials

The following supporting information can be downloaded at https://www.mdpi.com/article/10.3390/microorganisms11071774/s1, Table S1: Data used for statistic.
